# Do nasogastric or nasoenteric tubes improve outcomes from adhesional small bowel obstruction: a systematic review and meta-analysis

**DOI:** 10.1186/s12893-025-03207-x

**Published:** 2025-11-14

**Authors:** Ali Al-Mashat, Anaan Fareed, Tharindu Senanayake, Stephen Ridley Smith, Jonathan Gani

**Affiliations:** 1 Surgical Services, John Hunter Hospital, Newcastle, NSW Australia; 2 School of Medicine and Public Health, University of Newcastle, Newcastle, NSW Australia; 3 Department of General Surgery, Calvary Mater Hospital, Newcastle, NSW Australia

**Keywords:** Nasogastric tube, Adhesional small bowel obstruction, Operative management

## Abstract

**Objectives:**

To compare outcomes of nasogastric (NGT) or nasoenteric tube decompression against no decompression in the non-operative management of adhesional small bowel obstruction (ASBO) using a systematic review and meta-analysis.

**Methods:**

Database searches up to February 2025 were conducted using Cochrane Library, EMBASE, MEDLINE and SCOPUS. Abstract screening and data extraction were performed by two independent reviewers. Patients aged 18 and above were included. Studies were excluded if they compared NGTs to long tube devices or if the primary aetiology of SBO was not adhesions. Quality appraisal was conducted using the Newcastle Ottawa Scale and meta-analysis was performed using RevMan Web Software.

**Results:**

Searches yielded 1442 studies, of which 4 met the inclusion criteria, comprising a total of 1219 patients undergoing non-operative management for ASBO. These were all retrospective cohort studies. Within these studies, a total of 732 patients had a nasogastric or nasoenteric tube inserted for ASBO while 487 patients were managed without one. NGT use had a non-significant trend toward increased operative intervention, with a pooled odds ratio of 2.58 (95% CI: 0.77 to 8.65; *p* = 0.09, I² = 82%). Three studies compared bowel resection rates; NGT use was not associated with a statistically significant increased risk of bowel resection (OR 2.31; 95% CI: 0.86–6.16; *p* = 0.10). All studies reported a longer length of hospital stay in the NGT group.

**Conclusions:**

The available evidence is sparse, limited in design and quality, and marked by high heterogeneity, making it insufficient to draw a definitive conclusion regarding the role of NGTs in ASBO. High-quality evidence from a randomised controlled trial is needed to guide future practice.

**Trial registration:**

PROSPERO (CRD: CRD42021256098).

**Supplementary Information:**

The online version contains supplementary material available at 10.1186/s12893-025-03207-x.

## Introduction

Adhesive small bowel obstruction (ASBO) accounts for approximately 74% of small bowel obstruction cases, with its incidence continuing to rise [1, 2]. ASBOs are often managed non-operatively, except in cases with peritonitis, ischaemia, or strangulation. Non-operative management has been shown to be effective in approximately 70–90% of ASBO patients [3]. In contrast, SBOs caused by hernias, malignancies, strictures, or foreign bodies frequently require surgical intervention. Surgical management is associated with greater morbidity and mortality in ASBO patients, particularly if delayed [4, 5]. Furthermore, according to the Royal Australasian College of Surgeons Collaborative Hospitals Audit of Surgical Mortality (CHASM) data, small bowel resection for obstruction is the third leading cause of operative deaths [6]. Thus, the main priorities in ASBO management are early recognition of patients requiring surgical intervention and, when possible, timely resolution through non-operative management.

The cornerstone of non-operative management in ASBO has traditionally been the ‘drip and suck’ approach, involving intestinal decompression via a nasogastric (NGT) or nasoenteric tube [7], as recommended by the Bologna Guidelines from the World Journal of Emergency Surgery. Water-soluble contrast administration is a well-established intervention in the non-operative management of ASBO, with high-quality evidence supporting its diagnostic and therapeutic benefits, including reducing the need for surgery [8–10]. In contrast, despite its long-standing use, the value of NGT decompression remains unclear. Recently, a national survey of Australian surgeons highlighted controversy with its application and a shift towards its selective use in ASBO [11]. Given the significant risks and patient discomfort associated with NGT insertion [12, 13], a review of the evidence surrounding its use is needed to guide future practice and improve outcomes in patients with ASBO.

## Methods

### Research question

Does NGT or nasoenteric tube decompression improve outcomes in adults with ASBO?

### Protocol and registration

A protocol was designed in accordance with the Preferred Reporting Items for Systematic Review and Meta-analysis (PRISMA) 2020 checklist [14], and was registered prospectively under the PROSPERO systematic review database registration, CRD: CRD42021256098.

### Eligibility criteria

Studies were selected according to predefined inclusion and exclusion criteria.

Inclusion Criteria:

- Studies involving adult patients (≥ 18 years).
- Studies where the population included patients with ASBO, with a specified prior history of abdominal surgery.
- Studies comparing nasogastric or nasoenteric (e.g. long-tube) decompression against no decompression.
- Studies reporting outcomes including operative intervention, bowel resection or bowel necrosis, length of hospital stay, time to resolution, respiratory complications (including pneumonia), mortality, and resolution with water-soluble contrast administration.

Exclusion Criteria:

- Studies involving paediatric populations (< 18 years).
- Studies in which SBO was primarily due to causes other than adhesions (e.g., malignancy, hernias, inflammatory bowel disease or paralytic ileus).
- Studies focused on ASBO presentations requiring immediate surgical intervention.

For the purposes of this review, the “NGT group” refers to patients who received either a nasogastric or nasoenteric tube. Both forms of tube were considered functionally equivalent for decompression and were grouped together for analysis. In current practice, long tubes are less commonly used due to the complexity of their insertion. Although anatomical and mechanical differences exist between the two, their clinical effectiveness appears comparable; one study reported higher resolution rates with long tubes [15], while another showed no difference [16]. Therefore, any advantage associated with long tubes would likely bias results in favour of the NGT group.

Outcomes assessed:

- Operative intervention
- Bowel resection rates
- Mortality rates
- Incidence of pneumonia
- Length of hospital stay
- Time to oral intake
- Time to surgery
- Incidence of vomiting
- Success rate of water-soluble contrast challenge
- Quality of life

### Information sources and search

Electronic searches of the Cochrane Library, MEDLINE, EMBASE, and SCOPUS databases were performed in May 2023 and updated in February 2025. The search strategy was developed in collaboration with the John Hunter Hospital Library and incorporated MeSH terms and relevant synonyms. A full record of the search strategy is provided in the supplement (Appendix 1). The search targeted randomised controlled trials (RCTs), cohort studies, case-control studies, and other relevant observational designs, including prospective, retrospective, and cross-sectional studies. Case series and other study types were also considered. Abstract-only studies, opinion pieces, proposals, and animal studies were excluded. The search was restricted to English-language articles to ensure accurate interpretation of methodology and outcomes and to minimise the risk of data extraction errors. While this approach aims to enhance reliability, it may introduce language bias, particularly for a globally managed condition like ASBO, and could have led to the exclusion of relevant non-English studies. Reference lists of all included studies were examined to identify any additional eligible studies not captured in the initial database search. However, no grey literature databases were searched.

### Selection process and data extraction

After duplicate removal, all studies were screened for eligibility by title and abstract using Covidence, with access provided by the University of Newcastle. Two independent reviewers (A.A. and T.S.) conducted the initial abstract screening, with discrepancies resolved by a third reviewer (S.S.). Full texts of eligible studies were then retrieved and assessed by the same two reviewers. For the updated search, abstract and full-text screening was performed by A.A. and A.F. Data on study characteristics, population demographics, interventions, and outcomes were independently extracted by A.A. and A.F. into an Excel spreadsheet using a pre-defined data extraction template. The extracted data were cross-checked by both reviewers for accuracy.

### Study risk of bias assessment

Two authors (A.A. and A.F.) independently assessed the quality of the included studies using the Newcastle–Ottawa Scale (NOS). The NOS was selected for its applicability and widespread use in assessing the quality of non-randomised studies included in systematic reviews [17]. The NOS evaluates studies based on three domains: selection of study groups, comparability of groups, and ascertainment of exposure for cohort studies [17]. Each study was scored according to predefined criteria within these domains. Discrepancies in scoring were discussed and resolved through consensus, with input from a third reviewer (S.S.) when necessary.

### Certainty assessment (GRADE)

The Grading of Recommendations Assessment, Development and Evaluation (GRADE) approach was used to assess the overall certainty of evidence for each outcome in the systematic review, with ratings classified as high, moderate, low, or very low.

### Effect measures and data synthesis

For all dichotomous outcomes, effect estimates were calculated using the Mantel–Haenszel method and reported for both fixed-effect and random-effects models. For random-effects analyses, Hartung–Knapp–Sidik–Jonkman (HKSJ) method was applied when ≥ 3 studies were included and heterogeneity was greater than zero, to provide more conservative confidence intervals. For outcomes with only two studies or when heterogeneity was estimated as zero, Wald‐type confidence intervals were used. Outcomes with substantial heterogeneity (I² > 50%) were primarily interpreted using the random-effects results, whereas outcomes with low heterogeneity (I² < 50%) were primarily interpreted using the fixed-effect results. Presenting both models allowed assessment of how statistical heterogeneity influenced effect estimates. Odds ratios (ORs) with 95% confidence intervals (CIs) were generated. The statistical significance of the overall effect was assessed using either a Z-test (fixed-effect) or a T-test, with a p-value of less than 0.05 considered statistically significant. Results were also presented using forest plots. Anticipated absolute effects (AAE) and the summary of findings table were formulated using the GRADEpro Guideline Development Tool (GDT) [18]. 

For continuous outcomes, the initial plan was to calculate mean differences with 95% CIs. However, due to incomplete reporting, with missing standard deviations, a meta-analysis of continuous data was not feasible. These outcomes were instead summarised using descriptive analysis and narrative synthesis.

Attempts were made to obtain missing data from the corresponding authors from the relevant studies; however, no responses were received. Although methods for estimating means and standard deviations from medians and ranges were considered to allow for quantitative analysis, this was not feasible due to the small number of included retrospective studies, limited sample sizes, and inconsistent reporting of summary statistics. These limitations would have made any imputation unreliable and risked introducing bias, so a narrative synthesis was deemed more appropriate.

Statistical heterogeneity was assessed using the Chi squared (χ²) test and the I² statistic. An I² value above 50% was considered to indicate high heterogeneity. Statistical analyses were performed using RevMan Web software [19] which was also used to generate forest plots and perform meta-analyses. A subgroup analysis was conducted, restricted to studies with ASBO-only populations.

## Results

### Studies selection and study characteristics

**Fig. 1:**
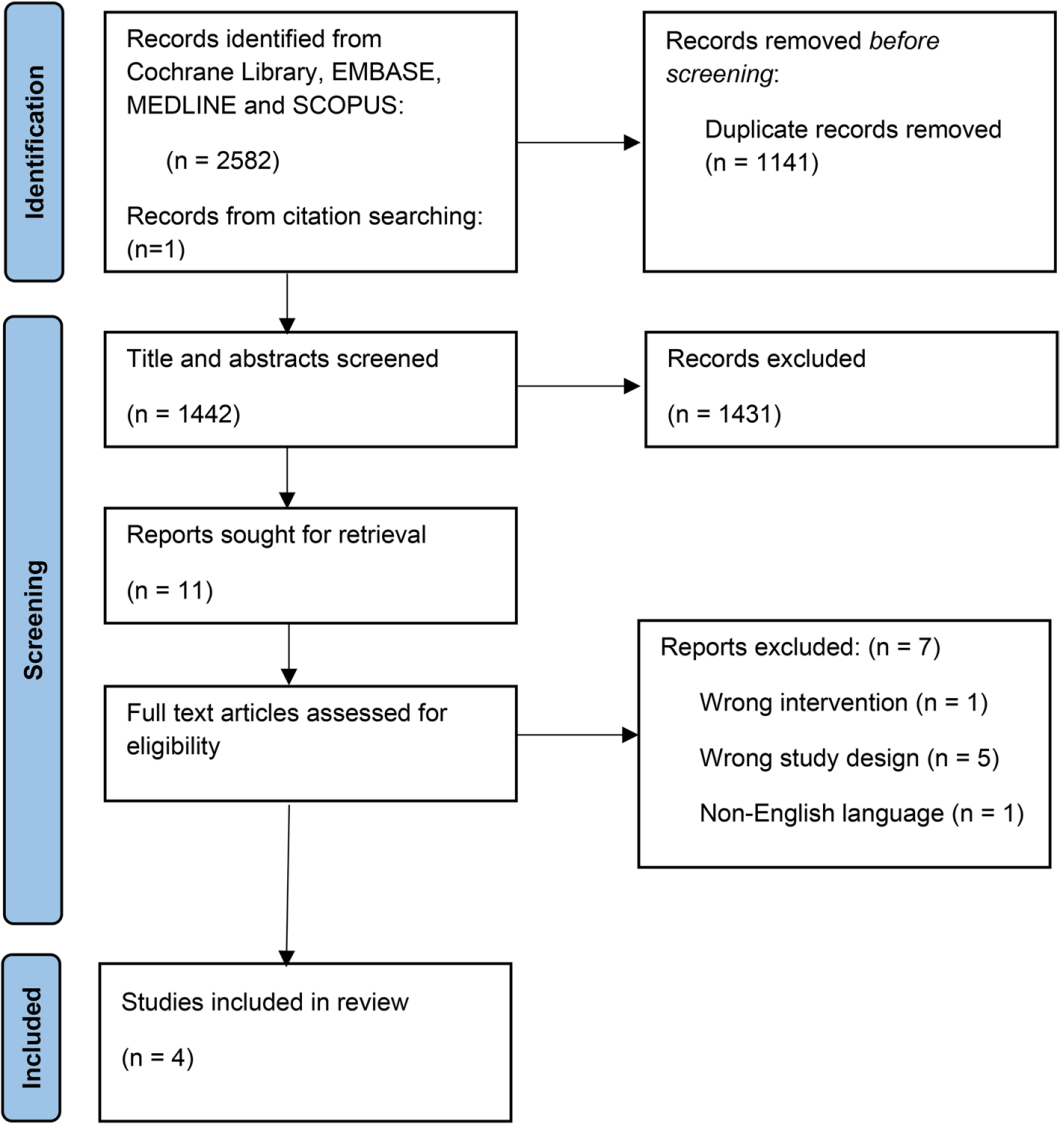
PRISMA flow diagram of study selection for inclusion in systematic review and meta-analysis of NGT use in ASBO

The initial search conducted in May of 2023 screened 1288 abstracts, resulting in the inclusion of three studies, one of which was identified through citation searching. The updated February 2025 search screened 1442 abstracts after duplicate removal and identified the same three studies along with one additional study (Fig. 1). Furthermore, one study (Berman et al.) [20] was included through citation searching. In total, 4 studies were included in the final analysis, comprising a total of 1219 patients undergoing non-operative management for ASBO, of which 732 were managed with a nasogastric or nasoenteric tube and 487 without one. Table 1 provides an overview of the characteristics of the included studies, along with their corresponding quality assessment scores.

**Table 1:** Study characteristics, risk of bias, and outcome definitions of included studies

	Fonseca et al.[21]	Berman et al.[20]	Shinohara et al.[22]	Al-Mashat et al.[23]
Year of publication	2013	2017	2022	2024
Country	United States of America	United States of America	Japan	Australia
Setting	Yale New Haven Hospital	Not specified	JA Shizuoka Kohseiren Enshu Hospital	John Hunter Hospital
Age (years)	NGT: mean = 58.71 No NGT: mean = 54.96	Median = 60 years	NGT: median = 76; range (17–96) No NGT: median = 69.5; range (30–102)	NGT: mean = 63.5 No NGT: mean = 59.4
Gender (% male)	NGT: 40.43% No NGT: 40%	NGT: 51% No NGT: 57%	NGT: 52.9% No NGT: 41.2	NGT: 52% No NGT: 48%
BMI (kg/m2) median	NR	NR	NGT: 19.7 No NGT: 20	NR
ASA scores	Not reported	Not reported	Not reported	Higher in the NGT group.
Sample size	290	181	288	460
Study design	Retrospective cohort	Retrospective cohort	Retrospective cohort	Retrospective cohort
Dates of study	Jan 2005 – Jun 2010	Sep 2013 – Sep 2015	Apr 2013 – Aug 2019	Jan 2016 – Dec 2020
Inclusion criteria	ICD-9 codes linked to ASBO, malignant SBOs, SBOs without clinically evident aetiology; >18 years; confirmed by CT or abdominal radiograph	ICD-9 codes linked to SBO; >18 years; CT confirmed SBO	ASBO confirmed on CT or radiograph; >18 years; history of previous abdominal surgery	> 18 years with ASBO (CT or clinical diagnosis); history of previous abdominal surgery
Exclusion criteria	Clinically evident incarcerated hernia; early post-op SBO (< 30 days); no radiological evidence; LBO; other causes	Early post-op SBO (< 4 weeks); non-adhesional SBO; ASBO requiring emergency surgery	ASBO requiring emergency surgery; non-adhesional SBO; palliated patients; other admissions	ASBO requiring emergency surgery; non-adhesional SBO; palliated patients; other admissions
Intervention	NGT (*n* = 235)	NGT (*n* = 93)	NGT (*n* = 88) or long tube (*n* = 52)	NGT = 264
Control	No NGT (*n* = 55)	No NGT (*n* = 88)	No NGT (*n* = 148)	No NGT (*n* = 196)
NGT protocol and adjunctive interventions	No information	No information	No information	Routine Gastrograffin^®^ use
Confounding adjustment method	Multivariate analysis and logistic regression	Multivariate analysis and logistic regression	None	None
Primary outcome	Factors associated with high NGT output	Operative rate; bowel resection; LOS; mortality	Incidence of vomiting	Operative rate
Secondary outcomes	Days to resolution; LOS; disposition; complications	Early predictors of surgery	Operative rate; mortality; LOS; time to oral intake; operative rate; pneumonia	Time to surgery; mortality; LOS; bowel resection
Newcastle Ottawa Scale score	8/9	8/9	7/9	7/9
Operative intervention definition	Not clearly defined	Surgical exploration with laparotomy	Not clearly defined	Not clearly defined
Bowel resection definition	Implied from intraoperative finding of bowel necrosis (not clearly defined)	Implied from intraoperative finding of bowel necrosis (not clearly defined)	Not assessed	Implied from intraoperative finding of bowel necrosis (not clearly defined)
Pneumonia definition	Pulmonary infiltrate on CXR or CT + clinical signs (fever, leukocytosis, sputum findings)	Not assessed	Presence of pulmonary infiltrates on CXR and ≥ 1 symptom (fever, purulent sputum, leukocytosis)	Not clearly defined
Mortality definition	Not assessed	In-hospital mortality	In-hospital mortality	In-hospital mortality
Declaration of conflict of interests	No information	Yes	Yes	Yes
Funding	No information	GWU Gill Fellowship	No information	None received

### Results of synthesis

#### Operative intervention

**Fig. 2:**
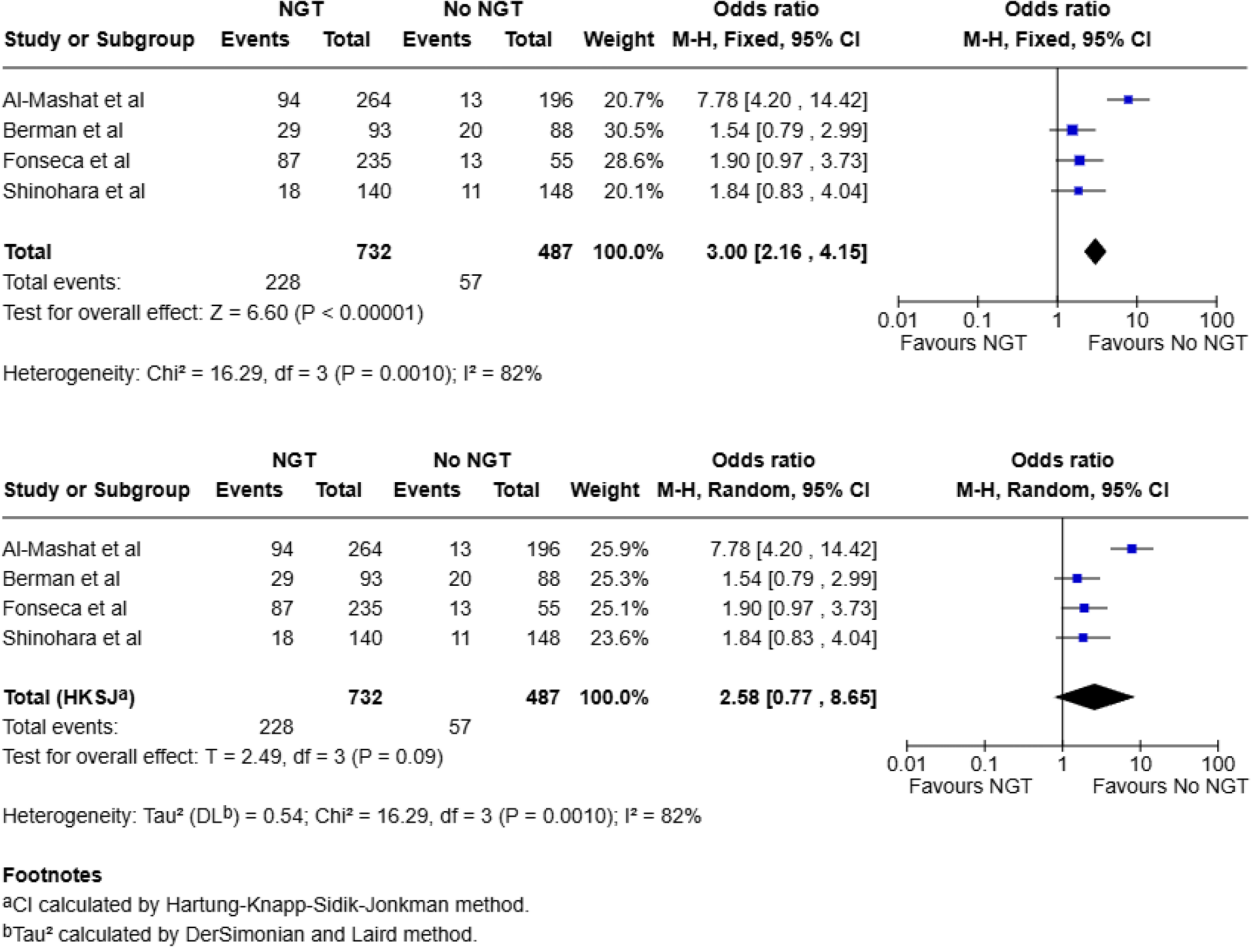
Meta-analysis for operative intervention using fixed and random-effects model

All four studies included data on operative intervention in NGT and no-NGT groups (total, 1219: NGT, 732; No NGT, 487) [20–23]. Of the 732 patients who had an NGT for conservative management of their ASBO, 228 (31.1%) underwent an operation, whilst 57 (11.7%) needed an operation in the no-NGT group. Meta-analysis using a random-effects model (Mantel–Haenszel method with HKSJ adjustment for confidence intervals) demonstrated a pooled odds ratio of 2.58 (95% CI: 0.77 to 8.65; *p* = 0.09), suggesting a non-significant trend toward increased operative intervention in the NGT group (Fig. 2). This outcome was associated with substantial statistical heterogeneity (I² = 82%, Tau² = 0.54), indicating considerable variability between studies. When a fixed effect model was applied, there was a statistically significant association between NGT use and increased odds of operative intervention (OR = 3.00, 95% CI 2.16–4.15, *p* < 0.00001; I² = 82%).

#### Bowel resection

**Fig. 3:**
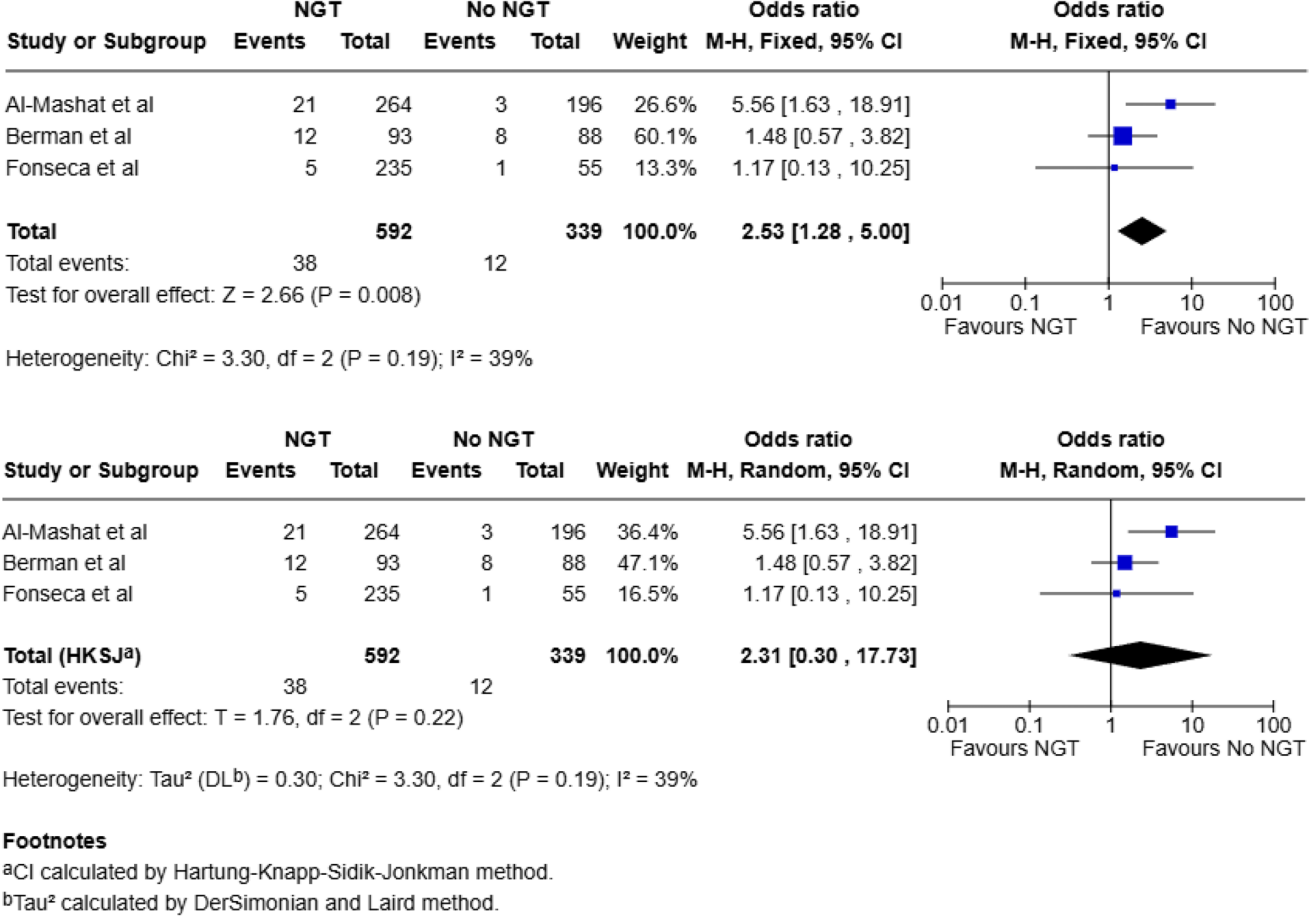
Meta-analysis for bowel resection using fixed effect and random-effects model

Three studies included intraoperative data on findings of bowel necrosis or rates of bowel resection (total, 931: NGT, 592; No NGT, 339) [20, 21, 23]. Of the 592 patients who had an NGT, 38 (6.4%) were found to have bowel necrosis or required a bowel resection compared to 12 of the 339 (3.5%) in the no-NGT group. Pooled analysis using a fixed-effect model demonstrated a statistically significant association between NGT use and increased risk of bowel resection (OR 2.53; 95% CI: 1.28–5.00; *p* = 0.008). When analysed using a random-effects model (HKSJ method), this association was no longer statistically significant (OR 2.31; 95% CI: 0.30–17.73; *p* = 0.22). There was moderate statistical heterogeneity (I^2^ = 39%) (Fig. 3).

#### Mortality

**Fig. 4:**
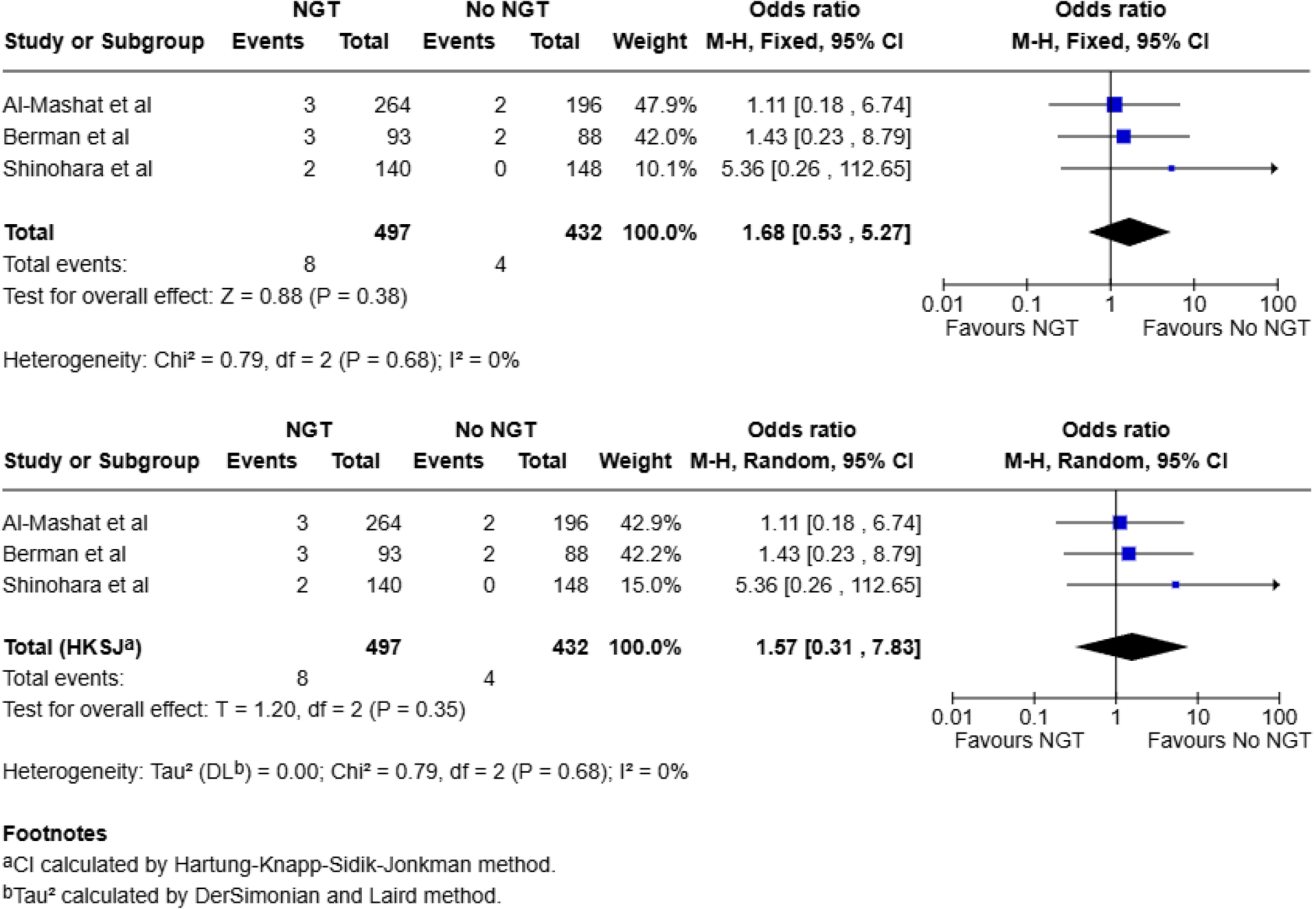
Meta-analysis for mortality using fixed effect and random-effects model

Three studies included data on mortality (total, 929: NGT, 497; No NGT, 432) [20, 22, 23]. A total of 12 deaths were reported across the three studies: 8 of 497 patients (1.6%) in the NGT group and 4 of 432 patients (0.9%) in the no-NGT group. Pooled analysis using a fixed-effect model showed no statistically significant association between NGT use and mortality (OR 1.68; 95% CI 0.53–5.27; *p* = 0.38). A random-effects model using the HKSJ method similarly showed no significant association (OR 1.57; 95% CI 0.31–7.83; *p* = 0.35). There was no evidence of statistical heterogeneity across the studies (I² = 0%, Chi² = 0.79, *p* = 0.68) (Fig. 4).

#### Incidence of pneumonia

**Fig. 5:**
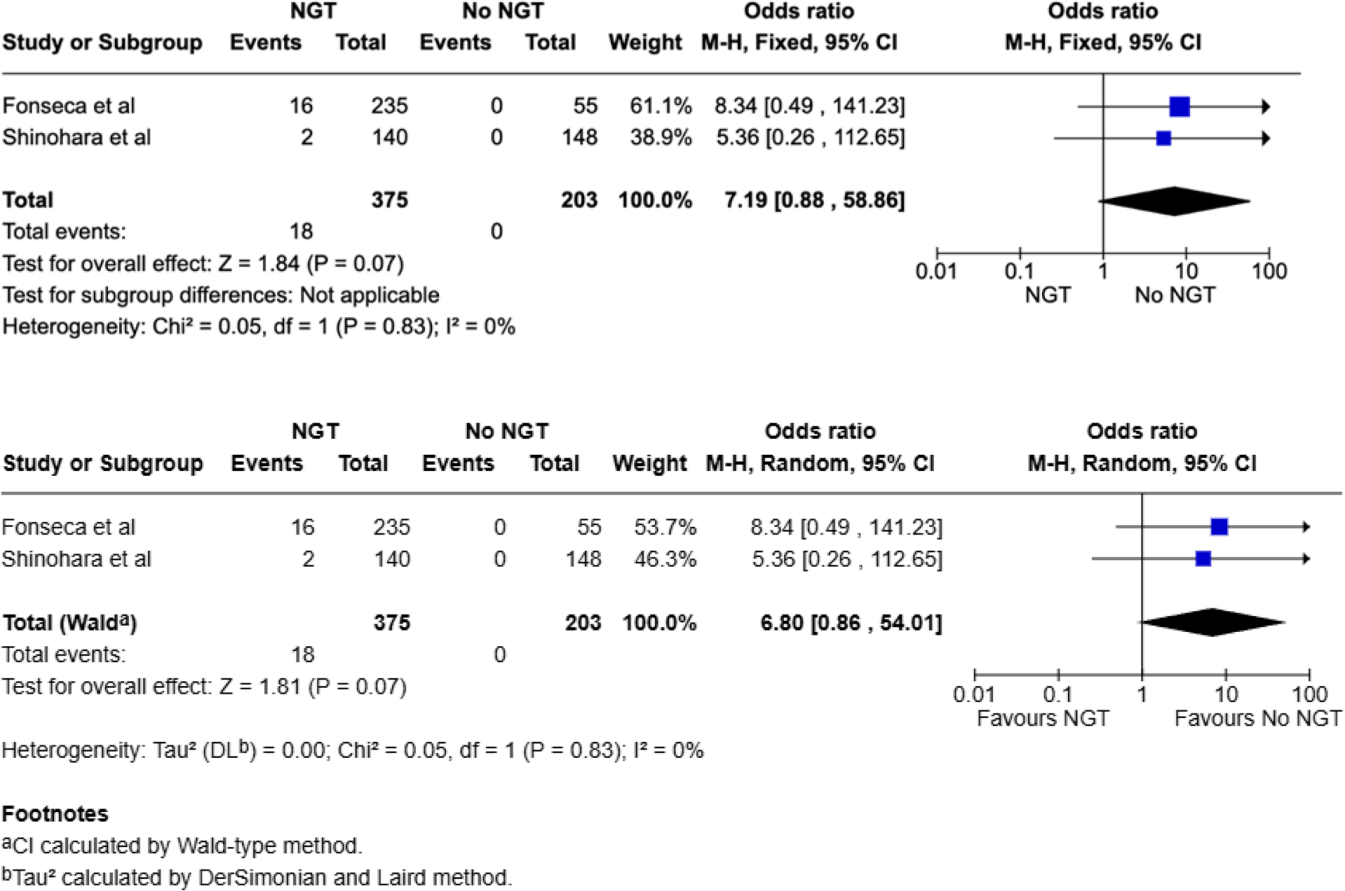
Meta-analysis for pneumonia using fixed effect and random-effects model

Two studies included comparative data on rates of pneumonia (total, 578: NGT 375; No NGT, 203) [21, 22]. There were 18 pneumonia cases in the NGT group (4.8%) and none in the no-NGT group. Pooled analysis using a fixed-effect model showed a non-significant trend toward increased risk of pneumonia with NGT use (OR 7.19; 95% CI: 0.88–58.86; *p* = 0.07). A random-effects model using the Wald-type method yielded similar findings (OR 6.80; 95% CI: 0.86–54.01; *p* = 0.07). There was no statistical heterogeneity (I² = 0%, Chi² = 0.04, *p* = 0.83) (Fig. 5). Although the point estimates suggest a potentially increased risk, the confidence intervals are wide and include the null, indicating imprecision. Interpretation is further limited by the absence of pneumonia events in the no-NGT group, which may have influenced the pooled estimates and reflects sparse event data.

#### Length of hospital stay

All four studies reported a longer length of hospital stay in the NGT group compared to the no-NGT group [20–23]. Shinohara et al. reported a median hospital length of stay of 10 days in the NGT group and 8 days in the no-NGT group [22]. Fonseca et al. reported a mean length of hospital stay of 10.16 days in the NGT group and 3.18 days in the no-NGT group [21]. Berman et al. reported a 7 day mean length of hospital stay in the NGT group and a 4.2 day mean in the no-NGT group [20]. Al-Mashat et al. reported a mean hospital length of stay of 6.9 days in the NGT group versus 3.2 days in the no-NGT group [23]. These results were not pooled due to incomplete reporting, including missing standard deviations and variation in how length of stay was reported across studies.

#### Time to oral intake

Two studies reported on time to oral intake [21, 22]. Shinohara et al. reported a median time of 5 days in the NGT group and 4 days in the no-NGT group [22]. Fonseca et al. reported the mean time of 3.55 days in the NGT group and 1.67 days in the no-NGT group [21]. 

#### Time to surgery

One study reported time to surgery, which was 58.8 h in the NGT group and 19.4 h in the no-NGT group [23]. 

#### Incidence of vomiting

One study reported the incidence of vomiting in ASBO patients after admission as 12.9% in the NGT group and 18.9% in the no-NGT group [22]. 

#### Success rate of water-soluble contrast challenge

No studies compared the rate of resolution with water-soluble contrast administration in NGT vs. no-NGT groups.

#### Patient reported outcomes and quality of life

No studies evaluated quality of life in patients managed with an NGT or nasoenteric tube compared to those without. Additionally, none assessed patient-reported outcomes such as pain, comfort, or overall experience.

#### Risk of bias within studies

The NOS scores by domain are shown in Table 2, with further detail on individual domain components provided in Appendix 2 (Table S1) of the supplementary material. Among the included studies, two studies received 8 out of 9 stars and two studies received 7 out of 9 stars. The higher quality studies were rated as being only somewhat representative of ASBO populations, due to their inclusion of patients with mixed aetiologies of SBO. The poorer quality ratings were assigned due to a lack of comparability and a failure to control for key confounding factors, which, for the purposes of assessing NGT as a therapeutic intervention in ASBO, were considered comorbidities and age. Given the quantity and nature of the eligible studies, formal assessment of publication and reporting bias, including the use of funnel plots, was not conducted.

**Table 2:** Newcastle Ottawa scale quality assessment scores

Study	Selection (Max 4)	Comparability (Max 2)	Outcome (Max 3)	Total Score (Max 9)
Fonseca et al.[21]	4	1	3	8
Berman et al.[20]	4	1	3	8
Shinohara et al.[22]	4	0	3	7
Al-Mashat et al.[23]	4	0	3	7

#### Quality of evidence

The summary of findings and corresponding GRADE certainty ratings are presented in Table 3, with footnotes detailing the reasons for downgrading or upgrading the quality of evidence for each outcome. The certainty of evidence for all outcomes ranged from low to very low and was downgraded due to reasons including the nature of the studies, relatively small sample sizes, limited adjustment for confounders, inconsistency due to heterogeneity, missing data sets and imprecision with large confidence intervals and lack of statistical significance. However, the certainty of evidence for operative intervention and bowel resection was upgraded from very low to low due to the large effect size. Length of stay was rated as low certainty, as the evidence came from observational studies showing consistent findings of longer admissions with NGT use, but lacked a pooled effect estimate.

**Table 3:** Summary of findings table: nasogastric or nasoenteric tube use versus non-use in ASBO

Outcome	Anticipated absolute effects* (95% CI)		Relative Effect (OR, 95% CI, *p*)*		Participants (Studies)	Certainty of Evidence (GRADE)
	No- NGT	NGT	Fixed effects	Random effects		
Operative intervention	117 per 1000	255 per 1000 (93 to 534)	OR 3.00 (2.16 to 4.15), *p* < 0.00001	OR 2.58 (0.77 to 8.65), *p* = 0.09	1219 (4 studies)	Low ⨁⨁⬤⬤^1^
Bowel resection	35 per 1000	85 per 1000 (45 to 155)	OR 2.53 (1.28 to 5.00), *p* = 0.008	OR 2.31 (0.30 to 17.73), *p* = 0.22	931 (3 studies)	Low ⨁⨁⬤⬤^2^
Mortality	9 per 1000	15 per 1000 (5 to 47)	OR 1.68 (0.53 to 5.27), *p* = 0.38	OR 1.57 (0.31 to 7.83), *p* = 0.35	929 (3 studies)	Very Low ⨁⬤⬤⬤^3^
Respiratory complications (Pneumonia)	0 per 1000	48 per 1000	OR 7.19 (0.88 to 58.86), *p* = 0.07	OR 6.80 (0.86 to 54.01), *p* = 0.07	578 (2 studies)	Very Low ⨁⬤⬤⬤^4^
Length of hospital stay	-	-	Not pooled; longer in NGT group		1219 (4 studies)	Low ⨁⨁⬤⬤^5^
Time to resolution (first oral intake)	-	-	Not pooled; longer in NGT group		578 (2 studies)	Very Low ⨁⬤⬤⬤^6^
Vomiting	-	-	Not pooled; lower in NGT group		288 (1 study)	Very Low ⨁⬤⬤⬤^7^
Time to surgery	-	-	Not pooled; longer in NGT group		460 (1 study)	Very Low ⨁⬤⬤⬤^8^
Success rate of water-soluble contrast challenge	-	-	Not pooled		0 studies	Very Low ⨁⬤⬤⬤^9^
Patient Reported Outcomes and Quality of life	-	-	Not pooled		0 studies	Very Low ⨁⬤⬤⬤^10^

*GRADE Working Group grades of evidence*

*High certainty: we are very confident that the true effect lies close to that of the estimate of the effect.*

*Moderate certainty: we are moderately confident in the effect estimate: the true effect is likely to be close to the estimate of the effect*,* but there is a possibility that it is substantially different.*

*Low certainty: our confidence in the effect estimate is limited: the true effect may be substantially different from the estimate of the effect.*

*Very low certainty: we have very little confidence in the effect estimate: the true effect is likely to be substantially different from the estimate of effect.*

*The risk in the intervention group (and its 95% confidence interval) is based on the assumed risk in the comparison group and the relative effect of the intervention (and its 95% CI). CI: confidence interval; OR: odds ratio; p: p value

^1^All included studies were retrospective and lacked adjustment for baseline differences, introducing risk of confounding. Substantial heterogeneity was present (I² = 82%), and the wide confidence interval (OR 2.58, 95% CI: 0.77–8.65) reflects imprecision. This suggests a possible increase in operative intervention with NGT use, with an estimated 138 more surgeries per 1,000 patients, though the certainty of this effect is low

^2^All studies were retrospective and at risk of confounding due to lack of statistical adjustment. Moderate heterogeneity was observed (I² = 39%), and the CI was wide (OR 2.31, 95% CI: 0.30–17.73), crossing the line of no effect. This suggests a possible increase in bowel resection with NGT use, with an estimated 50 more cases per 1,000 patients, though the evidence remains uncertain

^3^All studies were observational and unadjusted for confounding factors. Event rates were low, and the confidence interval was wide (OR 1.68, 95% CI: 0.53–5.27), crossing the line of no effect and indicating serious imprecision. This suggests a possible increase in mortality with NGT use with an estimated 6 more deaths per 1,000 patients, but the evidence is very uncertain

^4^Both studies were retrospective and unadjusted for confounding. All pneumonia cases occurred in the NGT group, with none in the control group, increasing uncertainty in the pooled estimate. The random-effects model yielded a high but imprecise odds ratio (OR 6.80, 95% CI: 0.86–54.01), with a wide confidence interval crossing the line of no effect. This suggests a possible increase in pneumonia with NGT use (48 more cases per 1,000 patients), though the certainty of this evidence is very low

^5^All studies reported longer hospital stays with NGT use. Certainty rated very low due to risk of bias, inconsistent reporting and missing data preventing meta-analysis

^6^Two studies reported longer time to oral intake in the NGT group. Certainty rated very low due to risk of bias, small sample sizes, inconsistent reporting (mean vs. median), and lack of pooled analysis due to missing data

^7^Based on a single retrospective study with risk of bias and no adjustment for confounding. Certainty rated very low due to study design limitations, imprecision, and lack of replication

^8^Reported by a single retrospective study without adjustment for confounding. Certainty rated very low due to study design limitations, imprecision, and lack of replication

^9^No studies reported comparative data on resolution rates following water-soluble contrast administration in NGT vs. no-NGT groups; therefore, the effect is not estimable, and the certainty of evidence is very low

^10^No studies assessed quality of life or patient-reported outcomes (e.g. pain, comfort, or overall experience) in patients with or without NGT use. Certainty rated as very low due to lack of evidence and inability to evaluate risk of bias, consistency, or precision

#### Sensitivity analyses

**Fig. 6:**
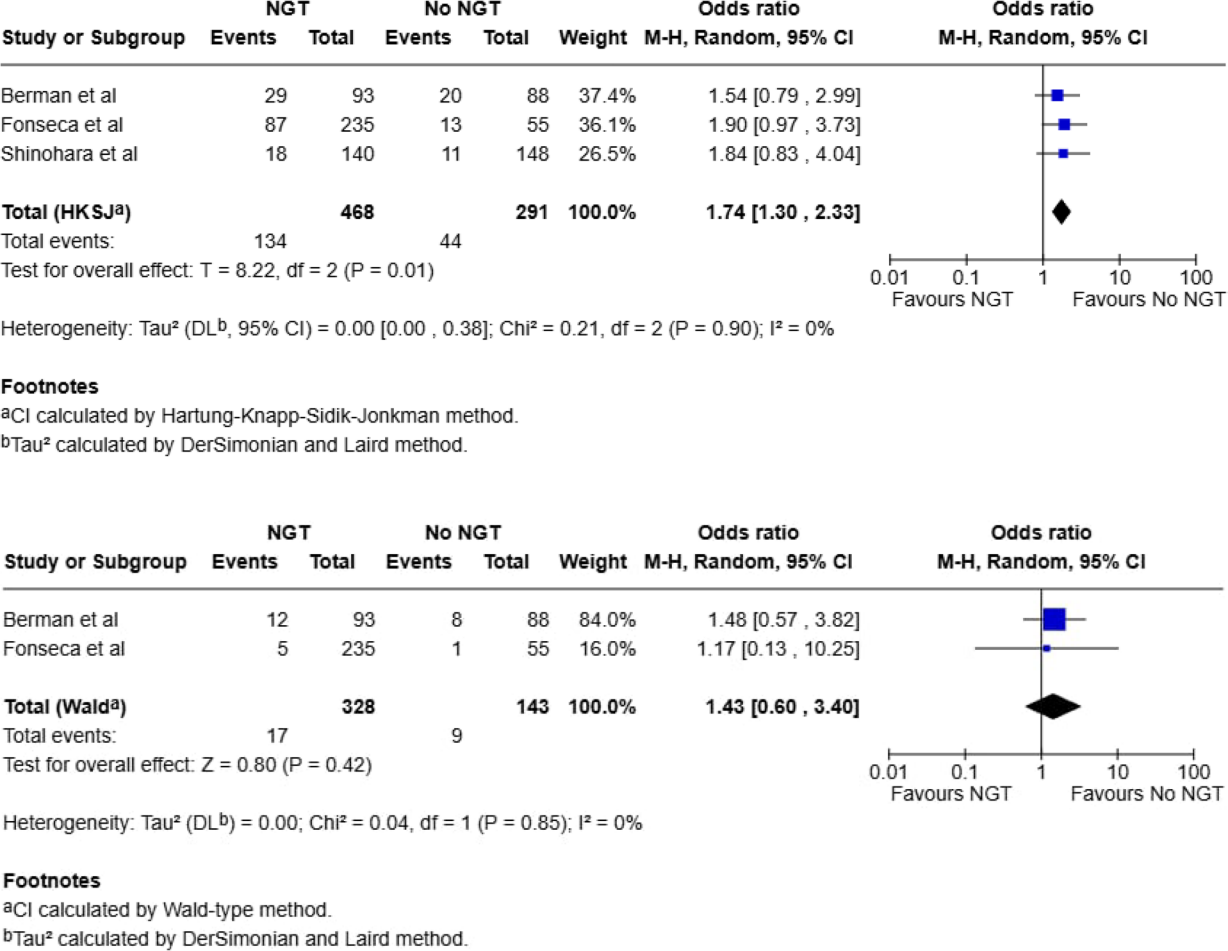
Leave-one-out sensitivity analysis for operative intervention (first) and bowel resection (second) using random-effects model with exclusion of Al-Mashat et al

**Fig. 7:**
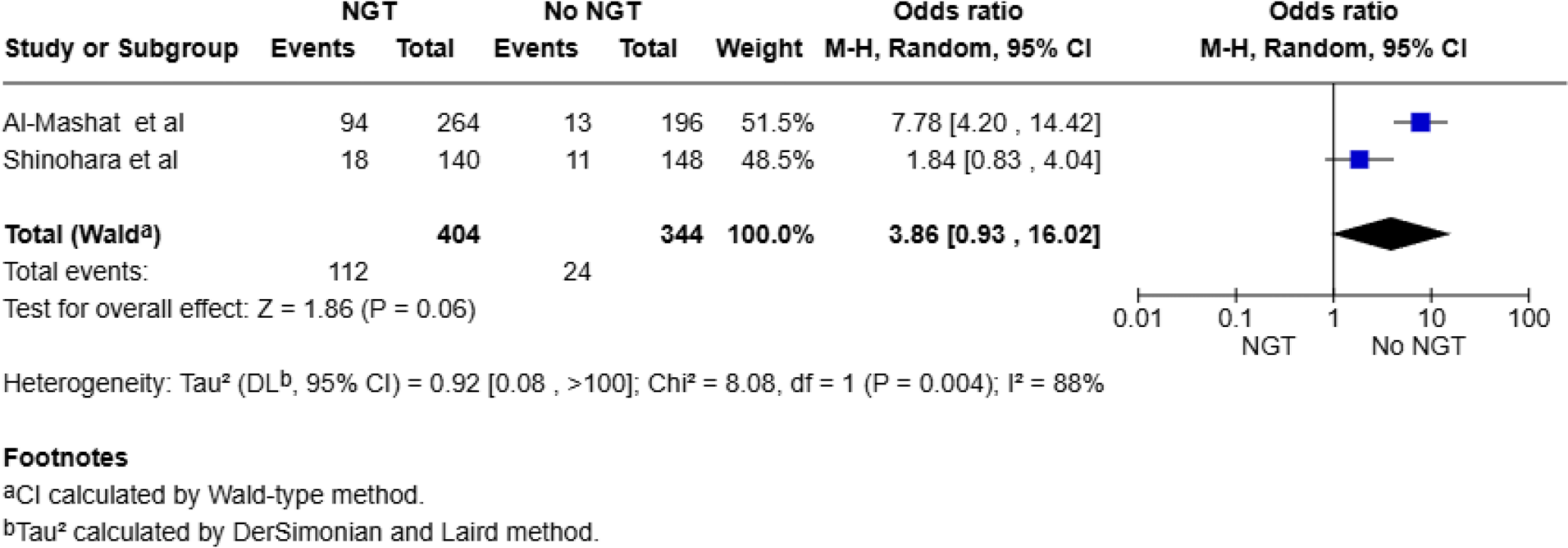
Subgroup analysis for operative intervention for ASBO-only studies using random-effects model

**Fig. 8:**
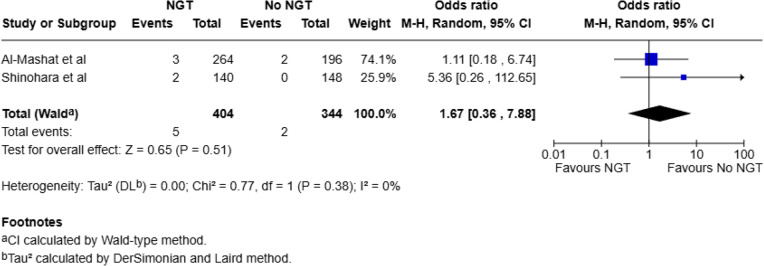
Subgroup analysis for mortality with ASBO-only studies using random-effects model

A leave-one-out sensitivity analysis was conducted for the outcomes operative intervention and bowel resection due to substantial heterogeneity. Exclusion of the study by Al-Mashat et al. [23] resulted in complete resolution of heterogeneity (I² = 0%) for the outcomes of bowel resection and operative intervention, suggesting this study was the primary source of statistical inconsistency, (Fig. 6). To assess the influence of including studies with non-adhesive SBO populations, we conducted a sensitivity analysis limited to studies exclusively reporting on ASBO (Al-Mashat et al. [23] and Shinohara et al. [22]). For operative intervention, the pooled OR was 3.86 (95% CI: 0.93–16.02; *p* = 0.06), with substantial heterogeneity (I² = 88%), (Fig. 7). For mortality, the pooled OR was 1.67 (95% CI: 0.36–7.88; *p* = 0.51), with no statistical heterogeneity (I² = 0%), (Fig. 8). While neither result was statistically significant, the mortality analysis suggested a potential trend toward increased risk in the NGT group. However, interpretation is limited by wide confidence intervals and small event counts. Sensitivity analyses for bowel resection and pneumonia were not possible, as both outcomes had only one study which reported them in an ASBO only population.

## Discussion

This review is based on four retrospective studies comparing nasogastric or nasoenteric tube use against its non-use in the management of ASBO. A key finding is the absence of prospective RCTs. Wangensteen, widely regarded as a prominent figure in the evolution of intestinal obstruction management and a proponent of enteric decompression, stated as early as the 1930s that the true value of this intervention could only be determined through clinical trials [24]. Our review highlights that, nearly a century later, prospective evidence remains unavailable. This is likely due to established surgical dogma, the perceived necessity of NGT insertion as an active intervention, and concerns that withholding it may be seen as substandard care. Moreover, the distress associated with ASBO may make patients and families hesitant to diverge from established practices, complicating recruitment and consent for trials that challenge conventional management.

Among the retrospective studies, pooled analyses using a random-effects model showed a non-significant trend toward higher rates of operative intervention and bowel resection in patients receiving nasogastric or nasoenteric decompression. In contrast, the fixed-effect model demonstrated a statistically significant association for both outcomes, an effect that was lost when heterogeneity was accounted for with the random-effects approach. This loss of significance reflects the random-effects model’s incorporation of between-study variability, which widens confidence intervals and reduces the dominance of larger studies, thereby attenuating the apparent effect observed under the fixed-effect assumption. For operative intervention, heterogeneity was high (I² = 82%), and moderate for bowel resection (I² = 39%), indicating variability between studies not accounted for by statistical modelling alone. This likely reflects a combination of statistical and clinical heterogeneity. Variability in study methodologies, particularly the failure to control for key confounding factors in Al-Mashat et al. [23] and Shinohara et al. [22] using approaches like multivariable regression or propensity score matching, may have further contributed to inconsistent effect estimates across studies. We did not perform meta-regression analyses due to the small number of included studies and limited sample sizes, which reduce statistical power and the reliability of the results. Furthermore, inconsistent reporting and limited availability of covariate data across the retrospective studies further limited such analysis. However, leave out sensitivity analysis for operative intervention and bowel resection showed that exclusion of the study by Al-Mashat et al.²³ reduced heterogeneity to zero, indicating that it was the primary source of variability.

Several clinical and methodological differences likely explain this effect. Al-Mashat et al. [23] reported a higher proportion of patients with elevated ASA scores in the NGT group, suggesting that tube placement was often a response to greater baseline illness severity rather than universal use. Such patients inherently have a higher likelihood of requiring operative intervention, introducing confounding by indication. When pooled with studies in which NGTs were placed more routinely or empirically across a broader spectrum of patients, this imbalance in baseline acuity amplified variability in effect estimates and contributed substantially to the high I² value observed. The study also differed from the others by reporting routine use of water-soluble contrast, which may have influenced both the timing of operative decisions (and whether bowel resection was required) and the likelihood of resolution without surgery. Furthermore, it was conducted in a high-volume Australian tertiary referral centre with a broad catchment area, likely resulting in an over-representation of more complex and severe cases. These factors make the study less directly comparable to others, explaining why its exclusion markedly reduced statistical heterogeneity. Although this heterogeneity was substantial, meta-analysis was still performed using a random-effects model to account for expected variability in study design and patient populations. The intent was to summarise existing evidence, explore trends, and highlight the need for higher-quality data, rather than to produce a definitive pooled estimate. Given this context, the pooled estimate should be interpreted with caution, as it may reflect underlying selection bias rather than a direct causal relationship between NGT use and surgical intervention. As such, the random-effects model provides the most appropriate summary, and as it demonstrated no statistically significant association with either operative intervention or bowel resection, no reliable conclusion can be drawn regarding a causal relationship between NGT use and these outcomes.

In addition to statistical heterogeneity, there was notable clinical heterogeneity among the included studies, arising from differences in setting, patient selection, management protocols, and outcome definitions. Geographical and temporal variation was present, with studies conducted across different countries, healthcare systems, and time periods ranging from the early 2000 s to 2020. Such variation likely reflects evolving clinical practice, surgeon preference, and institutional policy over time, which can alter both the likelihood of NGT placement and the threshold for operative intervention, thereby influencing effect estimates.

Patient acuity varied considerably between studies. In Al-Mashat et al., [23] NGT use was more frequent in patients with higher ASA scores, indicating a selective approach based on illness severity. In contrast, Fonseca et al., [21] reported a more empirical use of NGTs regardless of overall condition, although they found a positive association between NGT placement and diabetes. Higher baseline severity in the NGT group increases the probability of requiring surgery and bowel resection independent of NGT use, introducing confounding by indication that may bias results toward higher operative rates. Similar trends were observed in Berman et al., [20] where older patients and those with comorbidities or malignancy were more likely to receive an NGT.

Tube type was another differentiating factor. Shinohara et al., [22] conducted in Japan, used long tubes, a practice more common in East Asia [22, 25] and one that may have different clinical efficacy and complication profiles compared with standard NGTs, potentially altering rates of resolution and operative intervention. These tubes also differ from standard NGTs in their insertion methods, often requiring endoscopic decompression. This could potentially impact pneumonia rates, particularly if patients received sedation during the procedure and were at increased risk of aspiration. However, no information regarding sedation or aspiration risk was provided.

The aetiology of obstruction also differed. Fonseca et al.²¹ and Berman et al.²⁰ included non-adhesive causes of SBO, such as malignancy and hernia, which often require earlier surgical intervention and may increase operative rates independently of NGT use.

Outcome definitions were inconsistent. Only Berman et al.²⁰ explicitly defined operative intervention and restricted it to laparotomy, thereby excluding laparoscopic procedures. Other studies did not clarify whether laparoscopy was included. This inconsistency may have led to underestimation of operative rates in some studies and misclassification bias in pooled analyses.

Protocolised care differed between studies. Al-Mashat et al.²³ employed routine water-soluble contrast administration, which can be therapeutic in resolving ASBO as well as expedite diagnosis of non-resolving obstruction and prompt earlier surgery. Shinohara et al.²² did not use contrast, while other studies did not specify its use, leading to variability in both timing of intervention and non-operative resolution rates.

Each of these factors limits direct comparability between studies and may have influenced both the direction and magnitude of pooled estimates. While statistical heterogeneity was addressed through sensitivity and subgroup analyses, clinical heterogeneity remains a key limitation that may reduce the generalisability of findings. These results should therefore be interpreted with caution.

Narrative synthesis suggested that NGT decompression was consistently associated with longer hospital stays and delayed resolution, and in one study, with a prolonged time to surgery [23]. Köstenbauer et al. highlight the key challenge in ASBO management as the timely identification of patients with silent ischemia and propose the use of water-soluble contrast administered via an NGT as a method of accelerating recognition of failed conservative management [26]. These findings suggest that NGT decompression may, in practice, be failing to fulfil its intended therapeutic purpose, with potential adverse implications for patient outcomes and healthcare resource utilisation. However, the evidence is limited. All included studies were observational, with a high risk of confounding by indication, and missing data sets prevented robust pooling, thereby precluding definitive conclusions.

NGT use in ASBO is commonly justified by its perceived ability to reduce vomiting and prevent aspiration. This was also the most frequently selected reason for insertion in our survey of Australian surgeons [11]. There was only one study that reported on vomiting rates [22], and two on pneumonia rates [21, 22], with no significant association found. Logically, NGTs may reduce large-volume aspiration by emptying the stomach but can increase microaspiration risk by splinting open the oesophageal sphincters [26]. They may also provide symptomatic relief by reducing gastric distention, yet none of the studies in this review have assessed differences in patient comfort or quality of life. Our review of the literature demonstrates that there is no evidence that NGTs improve respiratory outcomes or reduce vomiting in ASBO patients. The need for a clinical trial evaluating this is crucial considering the risks of vomiting and aspiration NGTs carry just with insertion [12, 13]. 

The management of ASBO is complex and often guided by individual surgeon judgment and experience. Current SBO guidelines, including those from Bologna and Diaz [3, 27], recommend routine nasogastric decompression based predominantly on studies comparing nasogastric tubes with long intestinal tubes [16], or studies demonstrating acceptable rates of successful conservative management in patients receiving NGT decompression. However, these guidelines lack studies that directly evaluate NGT use as an intervention. As our review demonstrates, this is likely due to the scarcity of evidence specifically examining outcomes in ASBO patients managed without nasogastric decompression. To our knowledge, only one prior review, by Klingbeil et al., [28] has addressed this question. Our findings are consistent with theirs; however, our review provides updated evidence with a larger study pool and reinforces the lack of demonstrable benefit from NGT use. Nonetheless, as the quality of the included studies remains similar, this conclusion cannot be reliably stated, and further research is required.

### Strengths and limitations

A key strength of this review is its comprehensive search strategy, performed at two time points two years apart. It is also the largest systematic review to date addressing this question. The primary limitation is the reliance on retrospective cohort designs, which are inherently susceptible to selection bias and confounding which has downgraded the certainty of evidence across all outcomes. These studies cannot reliably account for clinical decision-making factors influencing NGT insertion, such as patient condition, symptom severity, perceived risk, and surgeon preference. As NGT use is often driven by these factors, it may have been preferentially used in sicker patients who differ systematically from those managed without NGTs. This introduces confounding by indication, which may explain the observed association with poorer outcomes. The reliance on retrospective studies, which are inherently unable to control for unmeasured confounders, weakens the strength of the conclusions of this review. As such, caution is warranted when interpreting these findings, and further high-quality research is needed to challenge conventional practice. Furthermore, the clinical heterogeneity between studies limits the generalisability of pooled results. Although inclusion of non-adhesional SBO was an intentional allowance due to the scarcity of ASBO specific data despite its clinical predominance, it may affect the applicability of these results to true ASBO populations. The review also grouped nasogastric and nasoenteric tubes despite their anatomical and potential functional differences, which may affect outcomes. This decision was made due to the limited number of eligible studies as separating the two modalities would have precluded meaningful synthesis and reduced analytical power. However, this approach may have contributed to clinical heterogeneity and introduced bias. Missing standard deviations also precluded quantitative synthesis for several outcomes, with the reliance on narrative synthesis weakening the overall strength of the evidence and introducing the potential for reporting bias.

### Implications for future practice

Due to the limitations of this review, no reliable conclusions can be made to guide the use of NGTs in ASBO. However, it emphasises the gap in high-quality evidence and supports the need for future research focusing on RCTs comparing NGT decompression versus no decompression in ASBO. Since it is possible that certain patients may benefit from decompression, future studies should also aim to identify patient factors to guide selective NGT placement and consider whether the combination of diagnostic and therapeutic contrast studies (i.e. Gastrograffin^®^ administration) with selective NGT use improves outcomes. NGT use should still be considered in ASBO management, however with the current state of knowledge, this data suggests that the apparent clinical need for NGT placement is a relative indication to consider the need for operative intervention instead.

## Conclusions

The outcomes of this review are significantly limited by the quality and strength of the available evidence and therefore do not allow for a definitive conclusion regarding the ability of nasogastric or nasoenteric tubes to reduce operative intervention rates or improve outcomes in ASBO. Further investigation in the form of a RCT is required.

## Supplementary Information

Supplementary Material 1.

Supplementary Material 2.

## Data Availability

Data is provided within the manuscript or supplementary information files.

## Abbreviations

ASBO: Adhesional small bowel obstruction
GRADE: Grading of Recommendations Assessment, Development and Evaluation
NGT: Nasogastric tube
NOS: Newcastle Ottawa Scale
NR: No record
OR: Odds ratio
CI: Confidence interval
LOS: Length of stay
