# Supplementary material for: Do nasogastric or nasoenteric tubes improve outcomes from adhesional small bowel obstruction: a systematic review and meta-analysis
Source: BMC Surg. 2025 Nov 14;25:545. doi: 10.1186/s12893-025-03207-x (PMC12619177; doi:10.1186/s12893-025-03207-x)
Supplement: Supplementary file 1 — Supplementary Material 1. [file 12893_2025_3207_MOESM1_ESM.doc]

**Database: Ovid MEDLINE(R) Epub Ahead of Print and In-Process, In-Data-Review & Other Non-Indexed Citations and Daily <February 26, 2025>**
**Search Strategy:**
**1**  exp Intestinal Obstruction/ (51748)
**2**  Tissue Adhesions/ (13852)
**3**  1 or 2 (63815)
**4**  exp Intestine, Small/ (170693)
**5**  3 and 4 (12721)
**6**  ((obstruct* or adhesi*) adj3 (small bowel or small intestin*)).ti,ab,kw,kf. (9137)
**7**  (aSBO* or SBO*).ti,ab,kw,kf. (2618)
**8**  5 or 6 or 7 (20255)
**9**  Intubation, Gastrointestinal/ (10459)
**10**  Decompression, Surgical/ (19033)
**11**  ((nasogastric or naso-gastric or nasoenteric or naso-enteric or nasointestinal or naso-intestinal orogastric or oro-gastric or small bowel or long) adj2 tube*).ti,ab,kw,kf. (9121)
**12**  ((gastrointestin* or gastro-intestin* or nasogastric or naso-gastric) adj2 intubat*).ti,ab,kw,kf. (685)
**13**  ((tube or nasogastric or naso-gastric) adj1 decompressi*).ti,ab,kw,kf. (629)
**14**  ((small bowel or small intestin*) adj3 tube*).ti,ab,kw,kf. (319)
**15**  (drainage adj3 tube*).ti,ab,kw,kf. (7095)
**16**  ((operativ* or surg*) adj3 (treat* or manag*)).ti,ab,kw,kf. (437267)
**17**  9 or 10 or 11 or 12 or 13 or 14 or 15 or 16 (475491)
**18**  Contrast Media/ (100199)
**19**  water soluble.ti,ab,kw,kf. (56206)
**20**  Diatrizoate meglumine/ (2207)
**21**  (meglumine adj1 diatrizoate).ti,ab,kw,kf. (442)
**22**  (gastrografin or urografin or MD-Gastroview or gastroview).ti,ab,kw,kf. (1014)
**23**  Fasting/ (39833)
**24**  (fast or fasted or fasting).ti,ab,kw,kf. (505045)
**25**  (bowel* adj2 rest*).ti,ab,kw,kf. (1085)
**26**  Conservative treatment/ (5730)
**27**  ((nonoperativ* or non-operativ* or conservativ* or nonsurg* or non-surg*) adj2 (treat* or manag*)).ti,ab,kw,kf. (116504)
**28**  Watchful waiting/ (5760)
**29**  (No-NGT or no-nasogastric).ti,ab,kw,kf. (58)
**30**  ((omitt* or omiss* or without) adj3 (nasogastric or naso-gastric*)).ti,ab,kw,kf. (130)
**31**  18 or 19 or 20 or 21 or 22 or 23 or 24 or 25 or 26 or 27 or 28 or 29 or 30 (789204)
**32**  8 and 17 and 31 (595)

**Database: Embase <1974 to 2025 February 26>**
**Search Strategy:**
**1**  small intestine obstruction/ (14696)
**2**  ((obstruct* or adhesi*) adj3 (small bowel or small intestin*)).ti,ab,kw,kf. (12837)
**3**  (aSBO* or SBO*).ti,ab,kw,kf. (3592)
**4**  1 or 2 or 3 (20911)
**5**  exp digestive tract intubation/ (7055)
**6**  decompression surgery/ (25089)
**7**  ((nasogastric or naso-gastric or nasoenteric or naso-enteric or nasointestinal or naso-intestinal orogastric or oro-gastric or small bowel or long) adj2 tube*).ti,ab,kw,kf. (13911)
**8**  ((gastrointestin* or gastro-intestin* or nasogastric or naso-gastric) adj2 intubat*).ti,ab,kw,kf. (818)
**9**  ((tube or nasogastric or naso-gastric) adj1 decompressi*).ti,ab,kw,kf. (1047)
**10**  ((small bowel or small intestin*) adj3 tube*).ti,ab,kw,kf. (400)
**11**  (drainage adj3 tube*).ti,ab,kw,kf. (10152)
**12**  ((operativ* or surg*) adj3 (treat* or manag*)).ti,ab,kw,kf. (571191)
**13**  5 or 6 or 7 or 8 or 9 or 10 or 11 or 12 (620109)
**14**  contrast medium/ (84018)
**15**  water soluble.ti,ab,kw,kf. (64919)
**16**  meglumine diatrizoate/ (4234)
**17**  (meglumine adj1 diatrizoate).ti,ab,kw,kf. (486)
**18**  (gastrografin or urografin or MD-Gastroview or gastroview).ti,ab,kw,kf. (1404)
**19**  fasting/ (30308)
**20**  (fast or fasted or fasting).ti,ab,kw,kf. (641729)
**21**  (bowel* adj2 rest*).ti,ab,kw,kf. (2074)
**22**  conservative treatment/ (114272)
**23**  ((nonoperativ* or non-operativ* or conservativ* or nonsurg* or non-surg*) adj2 (treat* or manag*)).ti,ab,kw,kf. (159758)
**24**  Watchful waiting/ (6227)
**25**  (No-NGT or no-nasogastric).ti,ab,kw,kf. (88)
**26**  ((omitt* or omiss* or without) adj3 (nasogastric or naso-gastric*)).ti,ab,kw,kf. (171)
**27**  14 or 15 or 16 or 17 or 18 or 19 or 20 or 21 or 22 or 23 or 24 or 25 or 26 (1009049)
**28**  4 and 13 and 27 (1009)

**Cochrane Library**

(((([mh "Intestinal Obstruction"]) OR ([mh ^"Tissue Adhesions"])) AND ([mh "Intestine, Small"])) OR (((obstruct* OR adhesi* ) NEAR/3 ("small bowel" OR ("small" NEXT intestin*) ))) OR ((aSBO* OR SBO* ))) AND (([mh ^"Intubation, Gastrointestinal"]) OR ([mh ^"Decompression, Surgical"]) OR (((nasogastric OR naso-gastric OR nasoenteric OR naso-enteric OR nasointestinal OR "naso-intestinal orogastric" OR oro-gastric OR "small bowel" OR long ) NEAR/2 tube* )) OR (((gastrointestin* OR gastro-intestin* OR nasogastric OR naso-gastric ) NEAR/2 intubat* )) OR (((tube OR nasogastric OR naso-gastric ) NEAR/1 decompressi* )) OR ((("small bowel" OR ("small" NEXT intestin*) ) NEAR/3 tube* )) OR ((drainage NEAR/3 tube* )) OR (((operativ* OR surg* ) NEAR/3 (treat* OR manag* )))) AND (([mh ^"Contrast Media"]) OR ("water soluble" ) OR ([mh ^"Diatrizoate meglumine"]) OR ((meglumine NEAR/1 diatrizoate )) OR ((gastrografin OR urografin OR MD-Gastroview OR gastroview )) OR ([mh ^Fasting]) OR ((fast OR fasted OR fasting )) OR ((bowel* NEAR/2 rest* )) OR ([mh ^"Conservative treatment"]) OR (((nonoperativ* OR non-operativ* OR conservativ* OR nonsurg* OR non-surg* ) NEAR/2 (treat* OR manag* ))) OR ([mh ^"Watchful waiting"]) OR ((No-NGT OR no-nasogastric )) OR (((omitt* OR omiss* OR without ) NEAR/3 (nasogastric OR naso-gastric* )))) 71 Results

**Scopus Basic**

(((("Intestinal Obstruction") OR ("Tissue Adhesions")) AND ("Intestine, Small")) OR (((obstruct* OR adhesi* ) W/3 ("small bowel" OR "small intestin*" ))) OR ((aSBO* OR SBO* ))) AND (("Intubation, Gastrointestinal") OR ("Decompression, Surgical") OR (((nasogastric OR naso-gastric OR nasoenteric OR naso-enteric OR nasointestinal OR "naso-intestinal orogastric" OR oro-gastric OR "small bowel" OR long ) W/2 tube* )) OR (((gastrointestin* OR gastro-intestin* OR nasogastric OR naso-gastric ) W/2 intubat* )) OR (((tube OR nasogastric OR naso-gastric ) W/1 decompressi* )) OR ((("small bowel" OR "small intestin*" ) W/3 tube* )) OR ((drainage W/3 tube* )) OR (((operativ* OR surg* ) W/3 (treat* OR manag* )))) AND (("Contrast Media") OR ("water soluble" ) OR ("Diatrizoate meglumine") OR ((meglumine W/1 diatrizoate )) OR ((gastrografin OR urografin OR MD-Gastroview OR gastroview )) OR (Fasting) OR ((fast OR fasted OR fasting )) OR ((bowel* W/2 rest* )) OR ("Conservative treatment") OR (((nonoperativ* OR non-operativ* OR conservativ* OR nonsurg* OR non-surg* ) W/2 (treat* OR manag* ))) OR ("Watchful waiting") OR ((No-NGT OR no-nasogastric )) OR (((omitt* OR omiss* OR without ) W/3 (nasogastric OR naso-gastric* )))) **Results:** **907**
