# Supplementary material for: Do nasogastric or nasoenteric tubes improve outcomes from adhesional small bowel obstruction: a systematic review and meta-analysis
Source: BMC Surg. 2025 Nov 14;25:545. doi: 10.1186/s12893-025-03207-x (PMC12619177; doi:10.1186/s12893-025-03207-x)
Supplement: Supplementary file 2 — Supplementary Material 2. [file 12893_2025_3207_MOESM2_ESM.docx]

| **Author** | **Year** | **Selection** | | | | **Comparability** | | **Outcome** | | | **Total score** |
| --- | --- | --- | --- | --- | --- | --- | --- | --- | --- | --- | --- |
|  |  | Representativeness of the exposed cohort | Selection of the non exposed cohort | Ascertainment of exposure | Demonstration that outcome of interest was not present at start of study | Comparability of cohorts on the basis of the design or analysis | | Assessment of outcome | Was follow-up long enough for outcomes to occur | Adequacy of follow up of cohorts |  |
| Fonseca et al.^21^ | 2013 | 1 | 1 | 1 | 1 | 1 | 0 | 1 | 1 | 1 | 8/9 |
| Berman et al.^20^ | 2017 | 1 | 1 | 1 | 1 | 1 | 0 | 1 | 1 | 1 | 8/9 |
| Shinohara et al.^22^ | 2022 | 1 | 1 | 1 | 1 | 0 | 0 | 1 | 1 | 1 | 7/9 |
| Al-Mashat et al.^23^ | 2024 | 1 | 1 | 1 | 1 | 0 | 0 | 1 | 1 | 1 | 7/9 |
